# Serum Metabolomics Analysis of the Anti-Inflammatory Effects of Gallic Acid on Rats With Acute Inflammation

**DOI:** 10.3389/fphar.2022.830439

**Published:** 2022-03-22

**Authors:** Yue Wu, Kuangyu Li, Maolin Zeng, Boyang Qiao, Benhong Zhou

**Affiliations:** ^1^ Department of Pharmacy, Renmin Hospital of Wuhan University, Wuha, China; ^2^ School of Pharmaceutical Sciences, Wuhan University, Wuhan, China; ^3^ Hubei No. 3 People’s Hospital of Jianghan University, Wuhan, China

**Keywords:** gallic acid, acute inflammation, metabolomics, protective effect, anti-inflammatory

## Abstract

**Background:** Gallic acid (GA) is a natural small-molecule polyphenol having a wide range of pharmacological activities. Until now, some works have studied the effect and the mechanisms of GA against inflammation. However, whether or how gallic acid regulates the downstream metabolic disorder against acute inflammation remains unclear. The present study explored the protective effect and the potential mechanism of GA on acute inflammation through the metabolomics approach.

**Methods:** An acute inflammation rat model was induced by local injection of carrageenin. Local swelling on paw and serum tumor necrosis factor-α (TNF-α), interleukin-6 (IL-6) were assessed in Control, Model and Gallic acid groups, respectively. Serum metabolomics based on high-performance liquid chromatography coupled with mass spectrometry (HPLC-MS) was also established to collect rats’ metabolic profiles and explore the metabolic changes related to GA pretreatment.

**Results:** Compared to the Modal group, local pain, redness, and swelling induced by carrageenin were significantly alleviated in GA groups in addition to the dose-dependent decreases of TNF-α and IL-6. Metabolomics analysis found significant alterations in metabolic signatures between the carrageenin-induced inflammation and control groups. Twelve potential biomarkers were further identified in acute inflammation by principal component analysis (PCA) and partial least squares discrimination analysis (PLS-DA). In addition, when rats were pretreated with gallic acid, serum levels of eleven biomarkers were observed to restore partially. Metabolic pathway and networks analysis revealed that GA might invert the pathological process of acute inflammation by regulating the key biomarkers involved in linoleic acid metabolism, ascorbate and aldarate metabolism, pentose and glucuronate interconversions, and arachidonic acid (AA) metabolism pathways.

**Conclusion:** The study elucidates the protective effect of gallic acid against acute inflammation and its possible regulating mechanism from a metabolomic perspective. These results could provide a theoretical basis for clarifying gallic acid’s mechanism and potential medicinal value in curing inflammation disorder in the clinic.

## Introduction

Inflammation is a common pathological phenomenon that plays a vital role in the human and animal disease spectrum. Excessive or inappropriate inflammatory responses are the basis of a series of pathological damage (Calder, 2006), resulting in various diseases, such as diabetes, atherosclerosis, rheumatoid arthritis, and some other life-threatening diseases (Pereira et al., 2011). In order to cure or prevent such diseases, anti-inflammatory regulation is now widely recognized and studied. The anti-inflammatory activities of natural ingredients are becoming increasingly attractive due to their relatively good safety properties and wide variety in nature.

Gallic acid is a well-known small-molecule polyphenol as the main component widespread in natural herbs such as pomegranate peel, Chinese gall, raspberry, and Radix Paeoniae Rubra (Zhang et al., 2018). As a natural antioxidant, gallic acid has a wide range of pharmacological activities, including antioxidant (Lambert and Elias, 2010), anti-inflammatory (Yoon et al., 2013; Seo et al., 2016), cardiovascular protection (Priscilla and Prince, 2009; Jin et al., 2018), bacteriostasis (Borges et al., 2013), hepatoprotection (Rasool et al., 2010; Hsieh et al., 2014) and anti-tumor activity (Nam et al., 2016; Pang et al., 2017). *In vitro*, several studies have certified the therapeutic effects of gallic acid on inflammatory diseases. Shin et al. and Kim et al. reported the activity of gallic acid in restraining the LPS-induced NO, PEG-2, and the production of interleukin-6 (IL-6) but with no cytotoxicity (Seo et al., 2016; BenSaad et al., 2017). Sripanidkulchai et al. studied the anti-inflammatory activity of the extract of the *Phyllanthus emblica Linn.* As the main component, gallic acid was able to inhibit the pro-inflammatory gene expression of cyclooxygenase-2 (COX-2), iNOS, IL-16, and IL-6 in a dose-dependent way (Sripanidkulchai and Junlatat, 2014). In addition, Mishra et al. reviewed gallic acid’s effect in inhibiting the activation NF- kappaB(NF-κB) and protein kinase B (Akt) signaling pathways along with the activity of some enzymes, such as cyclooxygenase, ribonucleotide reductase, thus preventing the occurrence of inflammatory tumors *in vitro* (Verma et al., 2013). *In vivo*, the protective effect of gallic acid on inflammatory damages has also been revealed in some diseases or disorders, like obesity (Tanaka et al., 2020; Gwon and Yun, 2021), chronic obstructive pulmonary disease (Singla et al., 2021), diabetes (Rahimifard et al., 2020), cisplatin nephrotoxicity (Dehghani et al., 2020), neuroinflammatory (Liu et al., 2020), colitis (Pandurangan et al., 2015; Zhu et al., 2019), and infection (Reyes et al., 2018). The main mechanisms include lowering the expression of inflammatory mediators (Reyes et al., 2018; Zhu et al., 2019; Tanaka et al., 2020; Singla et al., 2021), suppressing the phosphorylation or the metastasis of p65-NF-κB (Pandurangan et al., 2015; Zhu et al., 2019; Singla et al., 2021), inhibiting the activation of the signal transcription and transduction factor (Pandurangan et al., 2015), and downregulating mRNA and protein expression (Liu et al., 2020; Singla et al., 2021).

Although gallic acid’s activity against inflammatory diseases has been studied *in vitro* and *in vivo*, most of the works have focused on gene and protein expression mechanisms. Whether or how gallic acid regulates the downstream metabolic disorder in inflammation is unclear. At present, the relationship between inflammation and metabolic disorder attracts attention. Accumulating evidence indicates that metabolic disorders can trigger systemic inflammation, which in turn may play a role in pathophysiology or even aggravate the disease itself (Wang et al., 2015; Wenzl et al., 2021). Furthermore, recent studies also inspired that therapies targeting to restore metabolic homeostasis have the potential to treat inflammation damage (Zhuo et al., 2012; Lei et al., 2021). Therefore, studies focusing on the metabolic regulation of gallic acid may be valuable to explore the therapeutic effect and potential mechanisms of the candidate in the cure of inflammation disorder.

As one of the important branches of system biology (Fiehn et al., 2000), metabolomics is now a powerful technique to systematically characterize physiological and pathological changes of organisms (van der Greef et al., 2004). By studying the organisms’ changes of metabolic profile *in vivo* and exploring the relationship between metabolites and the physiological and pathological states, metabolomics has shown highly effective in investigating the physiological status of the body, diagnosing diseases, identifying perturbed pathways, as well as revealing the therapeutic effects, the material bases, and the mechanism of action of drugs (Huo et al., 2014; Wang et al., 2018). In this study, we explored the anti-inflammatory effect of gallic acid on acute inflammation through the serum biochemistry analysis and metabolomics approach, to revealing the therapeutic effects, the material bases, and the mechanism of action of drugs. LC-MS analysis was performed to study the serum metabolic profiles of rats with acute inflammation induced by carrageenan. The principal component analysis (PCA) and partial least squares discriminant analysis (PLS-DA) were conducted to investigate the changes of endogenous metabolites in order to evaluate the intervention effect of the gallic acid on inflammation. By identifying potential metabolic markers and analyzing metabolic pathways, the metabolic regulation of gallic acid was analyzed and discussed to provide insight into the systemic therapeutic effect of gallic acid on inflammation *in vivo*.

## Materials and Methods

### Materials

Gallic acid, aspirin, and carrageenin were purchased from Aladdin Chemistry Co., Ltd. (Shanghai, China) and Rhawn (Shanghai, China), respectively. The ELISA kits for the determination of TNF-α and IL-6 were obtained from Jiangsu Meimian industrial Co., Ltd. (Jiangsu, China). The Cleanert S C_18_ was obtained from Agela Technologies Co., Ltd. (Tianjin, China). The solvents used for LC-MS analysis were of chromatographic grade, and all other chemicals and solvents were of analytical grade.

### Animals

Forty-eight healthy male Sprague-Dawley rats (180 ± 20 g) were commercially obtained from Hubei Center for Disease Control and Prevention (Certificate NO. SCXK 2015-0018). Temperature and humidity were set at 21 ± 2°C and 60%. A regular 12 h light/dark cycle was established. All the rats were fed a standard diet with free access to water. The research did not include any human subjects. All animal experiments were approved by the ethics committee of Wuhan University, Wuhan, China, and carried out according to the National Institutes of health guide to care and use laboratory animals.

After acclimatization for 7 days, rats were randomly divided into six groups respectively containing eight rates, including normal control group (Control), acute inflammation Group (Model), high-dose gallic acid group (150 mg/kg, GAH), middle-dose gallic acid group (100 mg/kg, GAM), low-dose gallic acid group (50 mg/kg, GAL), and positive control group. Due to the effect of aspirin, a typical NSAID, against carrageenan-induced inflammation in previous reports, aspirin was selected as the control in positive control group (Aspirin) (Yao et al., 2015; Shui et al., 2016; Mahnashi et al., 2021). The Control and Model groups were gavaged with 2 ml of normal saline for three consecutive days, and Aspirin group was gavaged with aspirin (20 mg/kg/day) (Ma et al., 2020). In GAH, GAM, GAL groups, rats were administrated with gallic acid (150, 100, and 50 mg/kg/day, respectively) in the same way (Wei et al., 2018). Acute inflammation was induced 0.5 h after gavage on the third day. Rats in the group of Model, GAH, GAM, GAL, and Aspirin were subcutaneously injected with 0.1 ml of 1.0% carrageenan in the sub-plantar region of the right hind paws and the corresponding volume of normal saline in the Control group.

### Serum Sample Collection

Two hours after carrageenan injection, blood samples were collected from the abdominal aorta. Blood samples were centrifuged at 3000 rpm for 10 min, and the supernatant was collected and stored at-80°C for further use. One part of the serum sample was used to detect inflammatory factors TNF-α and IL-6 following the instructions of ELISA kits, and the other part for LC-MS metabolomics analysis.

### LC-MS Sample Preparation

The serum samples were thawed at room temperature prior to analysis. Firstly, 200 μL of methanol was added into 100 μL of the sample for protein-precipitation. The mixture was centrifuged at 8,000 rpm for 10 min and the supernatant were collected. The Cleanert S C_18_ was firstly activated by 5 ml methanol and 5 ml pure water, then the supernatant was added. Next, 5 ml pure water was used to elute, followed by 2 ml methanol. And the methanol eluent was collected for LC-MS analysis.

### HPLC-MS Analysis

HPLC-MS analysis was carried out using an HPLC-LTQ Orbitrap XL MS (ThermoFisher Scientific, Bremen, Germany) equipped with a C_18_ column (250 mm × 4.6 mm, 5 μm; ThermoFisher Scientific, Bremen, Germany) at 30°C. The mobile phase for the gradient elution was a mixture of water (A) and methanol (B) at a flow rate of 0.50 ml/min. A gradient program was as follows: 0–3 min: 10%B; 3–30 min: 10%-95%B; 30–40 min: 95%B; 40–55 min: 95%-10%B; 55–60 min: 10%B. The injection volume was 10 uL. The mass spectrometer was operated in negative ionization mode. The MS spectra were acquired from m/z 50 to m/z 1,000.

### Data Analysis

The data matrix, consisting of retention time and normalized peak area of metabolites, was imported into the SIMCA-P + 12.0 software (Umetrics, Sweden) and MetaboAnalyst 3.0 online software for multivariate data analysis, including pattern recognition based on PCA and PLS-DA. Potential biomarkers were selected according to the values of variable importance in the projections (VIP>1) through PLS-DA and the peak areas of different metabolites were further compared by *T*-test using SPSS 19.0 software for verification.

The databases such as Human Metabolome Database (HMDB) (http://www.hmdb.ca) and KEGG (http://www.kegg.jp/) were used for biomarker identification. The pathway analysis of potential biomarkers was performed with Metabolomics pathway analysis (MetPA) webserver software to identify the affected metabolic pathway analysis and visualization.

### Statistical Analysis

Pharmacodynamic data TNF-α and IL-6 levels were tested by one-way analysis of variance (ANOVA), and Student’s t-test tested differential metabolites levels. Data analysis was carried out by SPSS 19.0 software. The significant difference was considered at *p* < 0.05, and an extremely significant difference was considered at *p* < 0.01.

## Results

### Evaluation of the Anti-inflammatory

The Model group rats’ paws were swelling and fever 2 hours after carrageenan injection, indicating that acute inflammation models were established successfully. Compared with the Model group, the paw swelling degree decreased in GA groups, indicating that gallic acid could ameliorate the paw swelling induced by carrageenan in the rat model.

**FIGURE 1:**
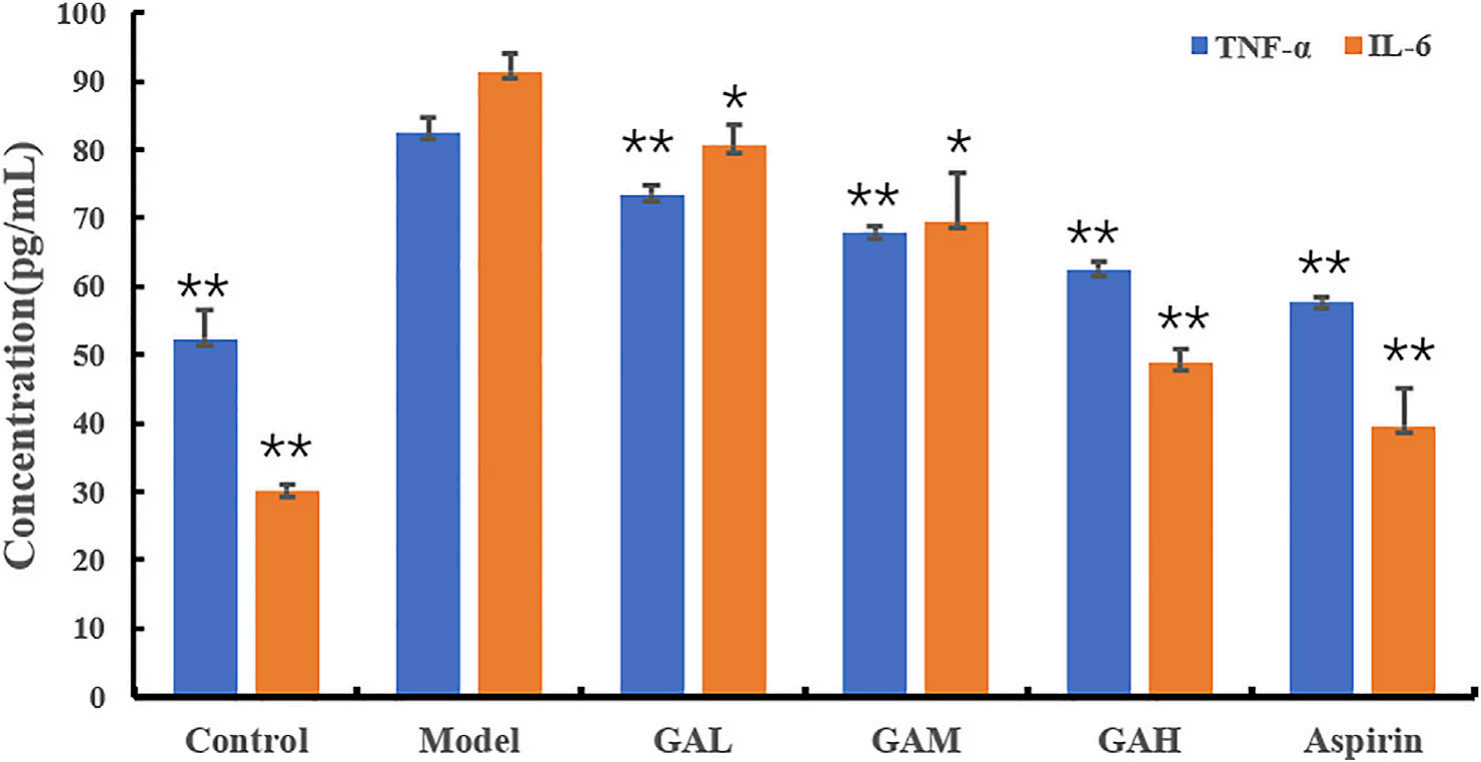
Effects of different doses of gallic acid on the levels of TNF-α and IL-6 (mean ± SD). Note: Significant differences were based on a two-tailed *t*-test. Compared with the Model group, *p < 0.05, **p < 0.01.

TNF-α and IL-6 are two of the typical inflammatory factors that play a vital role in inflammation. The inhibition effects of different doses of gallic acid on serum TNF-α and IL-6 were evaluated, and the results were shown in Table 1; Figure 1. Compared with the Control group, the levels of TNF-α and IL-6 were highly significantly (*p* < 0.01) increased in the Model group 2 h after carrageenan injection. It indicated that carrageenan-induced local inflammation stimulation triggered a systematic inflammatory response. Compared with the Model group, the serum TNF-α levels decreased highly-significantly (*p* < 0.01) in the GAH, GAM, and GAL groups, indicating that gallic acid was very effective in reducing TNF-α in serum. All doses of gallic acid could also lead to the decrease of IL-6 in serum. A significant difference (*p* < 0.05) was observed between Mode, GAL and GAM groups, and a highly significant difference (*p* < 0.01) was shown between GAH and Aspirin groups. Considering that middle-dose gallic acid showed a nice inhibition effect on the level of TNF-α and IL-6, the middle-dose gallic acid was used further to explore the anti-inflammatory mechanism through the metabolomics approach.

**TABLE1:** Effects of different doses of gallic acid on the levels of TNF-α and IL-6 (mean ± SD).

Group	TNF-α(pg·mL^−1^)	IL-6 (pg·mL^−1^)
Control	52.46 ± 4.26	29.78 ± 1.29
Model	82.24 ± 2.73^##^	90.97 ± 3.25^##^
GAL	73.55 ± 1.31**	80.27 ± 3.37*
GAM	67.55 ± 1.32**	69.08 ± 7.62*
GAH	62.47 ± 1.12**	48.43 ± 2.69**
Aspirin	57.89 ± 0.74**	39.23 ± 6.08**

Significant differences were based on a two-tailed *t*-test. Compared with the Model group, *p < 0.05, **p < 0.01; Compared with the Control group, ^#^ *p* < 0.05, ^##^ *p* < 0.01.

### Multivariate Data Analysis

**FIGURE 2:**
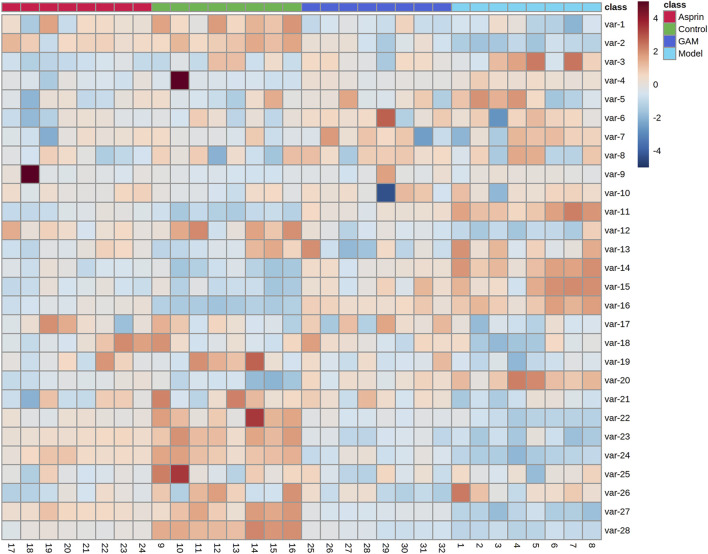
Changes of metabolites of control, model, aspirin, and middle-dose gallic acid group.

**FIGURE 3:**
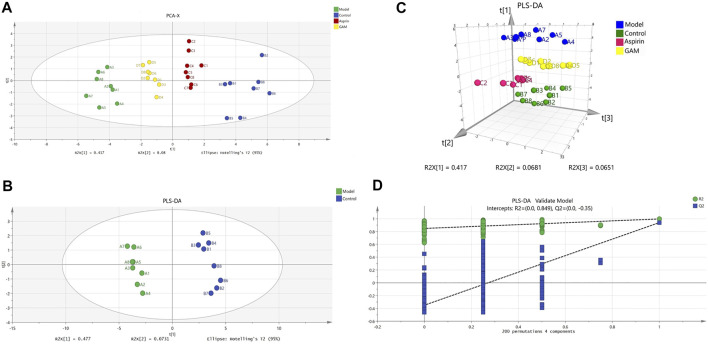
Scoring chart and 200 permutation tests (*n* = 8). PCA model of control, model, aspirin, and middle-dose gallic acid group **(A)**; PLS-DA model of control and model group **(B)**; Three-dimensional PLS-DA score plots of control, model, aspirin, and middle-dose gallic acid group **(C)**; Cross-validation of the PLS-DA model of control and model group (using 200 random permutations) **(D)**.

HPLC-MS/MS was used to characterize the metabolic profiles of serum samples in negative ion mode. Twenty-eight metabolites were found, and changes of metabolites were shown in the heatmap in Figure 2. The unsupervised PCA analysis on the data of different groups was performed to visualize general clustering, trends, or outliers among the observations. The PCA results were displayed as score plots, which represented the distribution of samples^[22]^. As shown in Figure 3A, the perfect separation of four groups was observed, indicating that each group had utterly different metabolic profiling. The most significant distance was found between the Control and Model group in PCA score plots, which reflected the perturbed metabolism in acute inflammatory and the successful establishment of the rat model. Plots of GA and Aspirin groups were situated between the Control and Model groups. It revealed that drug therapy could induce substantial and characteristic changes in metabolic profiles. In addition, like aspirin, a typical anti-inflammatory drug, pretreatment of gallic acid in rats changed metabolic profile from the state of disease to normal health, suggesting that gallic acid may ameliorate the physiological metabolism disorder of rats with acute inflammation.

### Identification and Analysis of Metabolites

In addition to the PCA analysis, a PLS-DA model was also established to search and identify the characteristic metabolic biomarkers associated with the inflammatory disorder in rat models (Figures 3B, C). Compared to PCA, the PLS-DA model is a supervised analysis that can better distinguish the difference between groups. After optimization, The quality parameters of the PLS-DA model between Control and Model groups were: R2X = 0.645, R2Y = 0.997, and Q2 = 0.94. Loading plots of PLS-DA were tested with 200 random permutations to assure reliability and guard against overfitting (Figure 3D).

**FIGURE 4:**
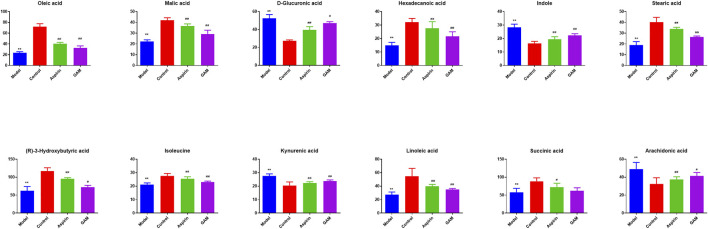
Potential biomarkers in serum with intervening of gallic acid and aspirin. The ordinate represents the numerical values of the metabolite biomarker with normalized peak areas (**p < 0.01 compared with the control; ^##^ *p* < 0.01, and ^#^ *p* < 0.05 compared with the model group).

Based on the VIP values (VIP>1) and correlation of Student’s t-test and *p* value (*p* < 0.05) in PLS-DA, twelve metabolic biomarkers were identified by comparing the metabolic profiles of the Control group with the Model. The details of these potential biomarkers are provided in Table 2; Figure 4. Compared to the Model group, eight identified metabolic biomarkers were significantly reduced in the Model group, while the remaining four markers were increased correspondingly. In addition, when the rats were pretreated with gallic acid, eleven of these metabolites (except succinic acid) showed a significant tendency to be normal, suggesting that gallic acid may fight against the inflammatory damage by regulating the disturbed endogenous biomarkers.

**TABLE 2:** Summary of potential metabolite markers.

No	Metabolites	R.T. (min)	m/z	Molecule Composition	VIP Value	Model	GAM
1	Oleic acid	39.297	282.2559	C_18_H_34_O_2_	1.496	↓**	↑^##^
2	Malic acid	40.009	134.0215	C_4_H_6_O_5_	1.483	↓**	↑^##^
3	D-Glucuronic acid	29.322	194.0426	C_6_H_10_O_7_	1.478	↑**	↓^##^
4	Hexadecanoic acid	38.79	256.2402	C_16_H_32_O_2_	1.458	↓**	↑^#^
5	Stearic acid	42.386	284.2715	C_18_H_36_O_2_	1.441	↓**	↑^##^
6	Indole	4.629	117.0578	C_8_H_7_N	1.438	↑**	↓^##^
7	(R)-3-Hydroxybutyric acid	3.421	104.0473	C_4_H_8_O_3_	1.389	↓**	↑^##^
8	Isoleucine	6.482	131.0946	C_6_H_13_NO_2_	1.361	↓**	↑^##^
9	Linoleic acid	37.607	280.2402	C_18_H_32_O_2_	1.319	↓**	↑^##^
10	Kynurenic acid	20.49	189.0426	C_10_H_7_NO_3_	1.315	↑**	↓^##^
11	Succinic acid	1.044	118.0266	C_4_H_6_O_4_	1.186	↓**	↔
12	Arachidonic acid	37.254	304.2402	C_20_H_32_O_2_	1.178	↑**	↓^#^

Compared with the Control group, *p < 0.05, **p < 0.01; Compared with the Model group, ^#^ *p* < 0.05, ^##^ *p* < 0.01.

### Metabolic Pathway Analysis

**FIGURE 5:**
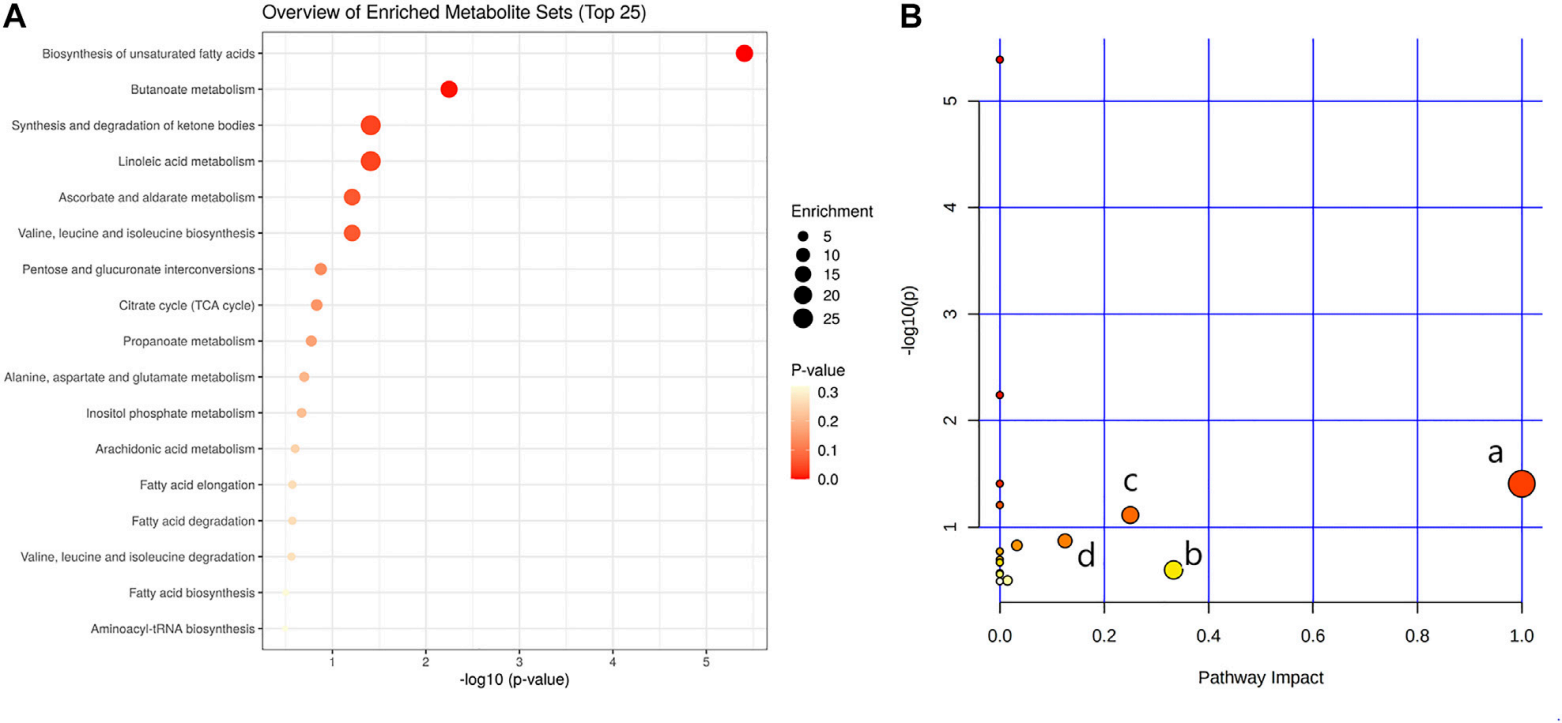
Pathway enrichment analysis **(A)** and metabolic pathway analysis **(B)**. a, linoleic acid metabolism; b, arachidonic acid metabolism; c, ascorbate and aldarate metabolisml; d, Pentose and glucuronate interconversions.

**FIGURE 6:**
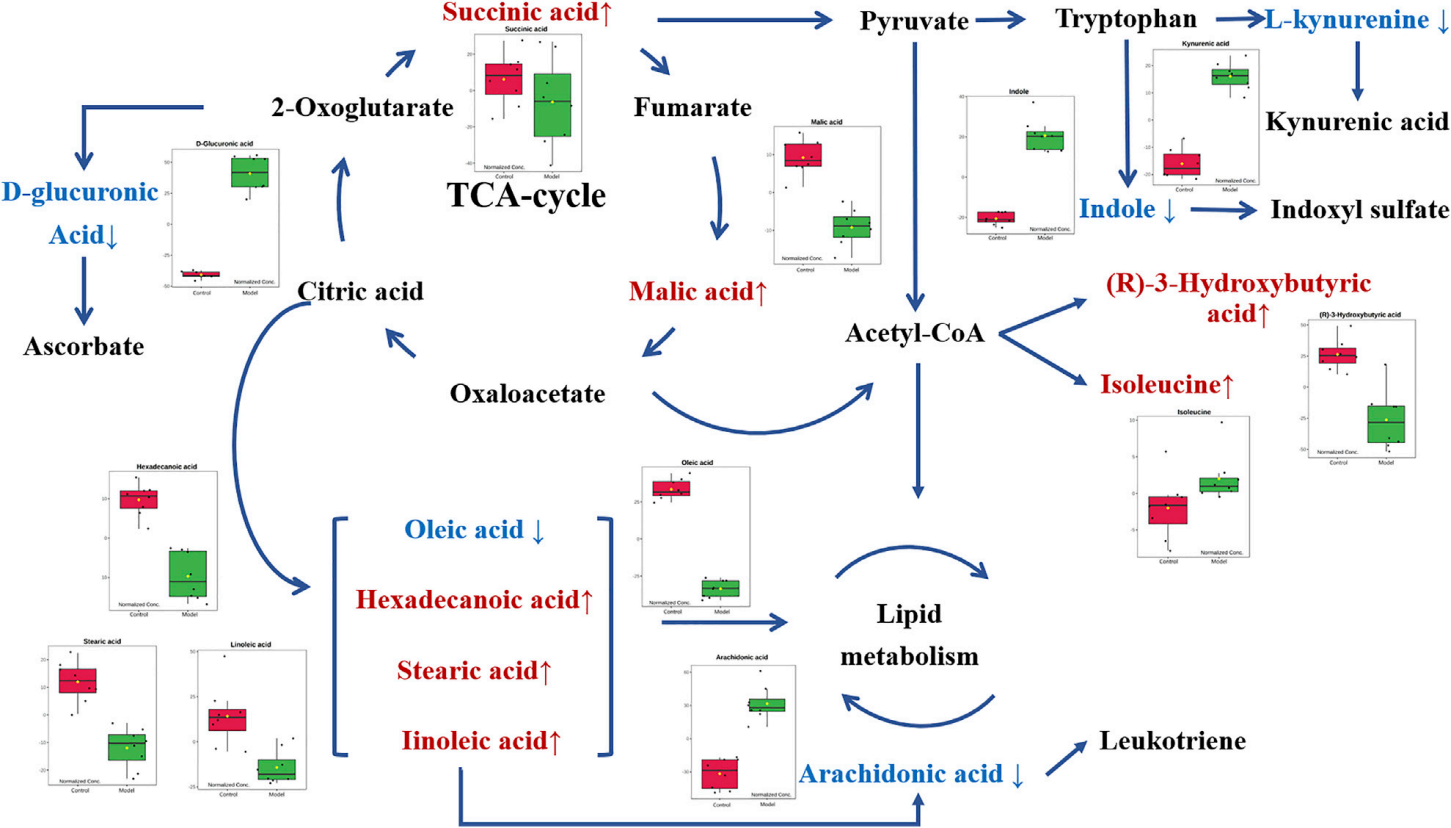
Metabolic networks of potential biomarkers in rat serum. Note: Metabolites in red and blue represent levels increased and decreased in the Model group, respectively.

In order to explore the potential mechanism and the regulatory network of the gallic acid’s anti-inflammation effect, twelve metabolic biomarkers were further analyzed through metabolic pathway analysis. The pathway enrichment analysis of endogenous differential metabolites was further performed by MetaboAnalyst 5.0. Seventeen metabolic pathways were found to be associated with carrageenin-induced inflammation in rats (Figure 5A). Four pathways with an impact value > 0.1 were considered to have the strongest correlation with the carrageenin-induced inflammatory disorder. These four pathways involved linoleic acid metabolism(impact value = 1.0), ascorbate and aldarate metabolism(impact value = 0.25), arachidonic acid metabolism(0.33292), and pentose and glucuronate interconversions (impact value = 0.125) (Figure 5B; Table 3). By referring to the existing literature and online databases such as KEGG and HMDB, the metabolic networks were structured and shown in Figure 6.

**TABLE 3:** Metabolic pathway enrichment table of rat serum.

NO.	Pathway Name	Total cmpd	Hits	RawP	-log(p)	Impact	Details
1	Biosynthesis of unsaturated fatty acids	36	5	4.0831E-6	5.389	0.0	KEGG
2	Butanoate metabolism	15	2	0.0057498	2.2403	0.0	KEGG
3	Synthesis and degradation of ketone bodies	5	1	0.039185	1.4069	0.0	KEGG
4	Linoleic acid metabolism	5	1	0.039185	1.4069	1.0	KEGG
5	valine, leucine and isoleucine biosynthesis	8	1	0.062015	1.2075	0	KEGG
6	Ascorbate and aldarate metabolism	10	1	0.076958	1.1137	0.25	KEGG
7	Pentose and glucuronate interconversions	18	1	0.13457	0.87104	0.125	KEGG
8	Citrate cycle_(TCA cycle)	20	1	0.14845	0.82841	0.03273	KEGG
9	Propanoate metabolism	23	1	0.16889	0.7724	0.0	KEGG
10	Alanine, aspartate and glutamate metabolism	28	1	0.20195	0.69475	0.0	KEGG
11	Inositol phosphate metabolism	30	1	0.21484	0.66789	0.0	KEGG
12	Arachidonic acid metabolism	36	1	0.25235	0.59799	0.33292	KEGG
13	Fatty acid metabolism	39	1	0.27049	0.56785	0.0	KEGG
14	Fatty acid elongation	39	1	0.27049	0.56785	0.0	KEGG
15	valine, leucine and isoleucine degradation	40	1	0.27645	0.55839	0.0	KEGG
16	Fatty acid biosynthesis	47	1	0.3169	0.49908	0.01472	KEGG
17	Aminoacyl-tRNA biosynthesis	48	1	0.32251	0.49146	0.0	KEGG

Total Compound: the total number of compounds in the pathway; hits: the matched number from the user uploaded data; RawP: the original *p*-value calculated from the enrichment analysis; Impact: pathway impact value calculated from pathway topology analysis.

## Discussion

Inflammation is an essential immune response that promotes the survival of a host in the presence of a variety of internal and external insults (Hunter et al., 2009). Nevertheless, excessive inflammation may destroy cells or disturb cellular metabolism, thus contributing to chronic diseases and even death. Anti-inflammatory regulation has become attractive in preventing or curing inflammation-associated disorders. Gallic acid is a well-known natural polyphenol with various pharmacological activities. Although some previous studies have demonstrated the mechanism of the anti-inflammation of gallic acid, its effect in regulating the metabolic disorder in inflammation is unclear. In this research, a metabolomics approach was applied to give a global view of the characteristic metabolic profile associated with the anti-inflammatory effect of gallic acid. It will provide insight into the systemic therapeutic effect of gallic acid in inflammation diseases and help elucidate its mechanisms of action.

A carrageenin-induced inflammation mice model was used to evaluate the effect of the anti-inflammation of gallic acid in the present study. Carrageenin is a polysaccharide vegetable gum obtained from Irish moss, which can induce a neutrophil-mediated acute inflammatory response when injected into the sub-plantar region of mice. This model is well-researched and highly reproducible, thus usually used to study acute inflammatory processes (Morris, 2003). In this research, after being injected with 0.1 ml of 1.0% carrageenan, paw edema with redness and swelling and elevated serum TNF-α and IL-6 were observed in the Model group, indicating that the acute inflammation model was successfully established. While in GA groups, the paw edema was significantly alleviated with gallic acid administration. Decreased levels of inflammatory cytokines TNF-α and IL-6 were also observed in serum biochemistry analysis and were dosa-dependent with gallic acid pretreatment. It demonstrated that the anti-inflammatory effect of gallic acid might be associated with the down-regulation of TNF-α and IL-6. These results were similar to the results reported by Tahereh et al. and Fan et al. (Setayesh et al., 2019; Zhu et al., 2019; Singla et al., 2020).

In the study of drug metabolomics, the isolation, purification, and identification of endogenous substances in biological samples are critical. Biological samples have the characteristics of complex components, low concentration of substances to be measured, and more interfering substances. Therefore, high sensitivity and high specificity analysis methods are required, and pretreatment such as separation and concentration is also undergone before the analysis. Solid-phase extraction (SPE) is an effective pretreatment technology that combines solid-liquid extraction and column liquid chromatography. Compared with traditional extraction, SPE can separate impurities more effectively, with shorter sample treat time and more convenient operation. In our study, C18 SPE was used to pretreat the serum samples, and the high-resolution Orbitrap MS was used to detect endogenous metabolites in serum samples.

As an overall framework, metabolomics characterizes the functional phenotype of a system under certain conditions through the low molecular weight metabolites. Through high-resolution analytical detection and multidimensional statistical analysis, metabolomics presents a global view of endogenous and exogenous metabolites to study the fundamental causes of diseases, drug toxicity or identify the biomarkers associated with the diagnosis or therapeutic efficacy. This study compared the serum metabolomics characteristics of normal rats and acute inflammation modeled rats using an HPLC-MS platform and multivariate data analysis. Compared with the control group, there was a significant metabolic disorder in the model group, indicating that local stimulation of carrageenin caused systemic inflammation in rats. Twelve relevant metabolites were then screened out by PCA and PLS-DA analysis, including oleic acid, malic acid, D-glucuronic acid, hexadecanoic acid, stearic acid, indole, 3r-hydroxy-butanoic acid, isoleucine, linoleic acid, canine urine, succinic acid, and arachidonic acid. In addition, when pretreatment of gallic acid was applied, eleven of these metabolites (except succinic acid) showed a significant tendency to be normal. These potential metabolites are mainly involved in linoleic acid metabolism, ascorbate and aldarate metabolism, pentose and glucuronate interconversions, and arachidonic acid metabolism. Most of these potential metabolites are directly or indirectly connected with each other.

In metabolic networks, lipid metabolism is closely related to inflammation (Kubala et al., 2010). Arachidonic acid (AA) is an essential fatty acid existing in the cell membrane in the form of phospholipids [29]. When the cells are under stress, especially in response to inflammatory stimulation, AA is released from the phospholipids and then oxidized or modified into various bioactive metabolites, including prostaglandins, thromboxanes, and leukotriene, thus promoting inflammatory cascades (Yao et al., 2015; Wang et al., 2019). *In vivo*, AA originates from direct dietary intake and the elongation-desaturation process of its precursor linoleic acid, but only the biological transformed AA is pro-inflammatory (Monteiro et al., 2013). In the present work, the decrease of serum linoleic acid was observed in the Model group along with the increase of arachidonic acid. It is consistent with the conclusion that inflammatory stimuli trigger the transformation of AA in the acute inflammation process. Moreover, with the intervention of gallic acid, serum linoleic acid tended to increase with the corresponding decrease of arachidonic acid, suggesting that gallic acid might act as an anti-inflammatory by regulating the transformation of AA *in vivo*.

D-glucuronic acid is one of the potential metabolic biomarkers that has been identified in inflammation (Dong et al., 2021; Olsson et al., 2021). Elevated D-glucuronic acid was found in the zebrafish model to be against the inflammatory response (Dong et al., 2021). Plasma D-glucuronic acid has also been reported to help the discrimination of patients with inflammation (Olsson et al., 2021). D-Glucuronic acid is a metabolite of glucose *in vivo*. It is formed from glucose by the multi-step metabolic process including citrate cycle, propanoate metabolism, pentose and glucuronate interconversions, and further converted to ascorbic acid or xylulose in ascorbic acid metabolism through a sequence of enzyme-driven steps (Liu et al., 2015). Ascorbic acid, known as vitamin C, is an ingredient with well-known antioxidant, anti-inflammatory, and immunomodulatory activities (Kianian et al., 2019). Ascorbic acid can inhibit the activation of the leukocyte myeloperoxidase/H2O2/Halide system, thereby improving leukocyte movement (Yara et al., 2013). In this study, the level of D-glucuronic acid increased in the Model group compared with the Control group, which is consistent with the previous reports (Dong et al., 2021; Olsson et al., 2021). It suggested that the carrageenin stimulated the release of more ascorbic acid to suppress inflammation. When the gallic acid intervention was applied, the serum level of D-glucuronic acid decreased, indicating that the medication of gallic acid can fight against the inflammatory response *in vivo*.

Isoleucine, leucine, and valine are essential branched amino acids that serve as substrates and signaling molecules to regulate protein synthesis in skeletal muscle. Skeletal muscle produces most of the body’s glutamine, the substrate for cell proliferation, including immune cells. In our study, when inflammation occurs, isoleucine content decreases, which may be caused by the consumption of isoleucine resulting from the proliferation of extensive inflammatory cells. With the pretreatment of gallic acid, the level of isoleucine increased, which may be that gallic acid inhibits the proliferation of inflammatory cells. In addition, the tryptophan (TRP)-kynurenine (KYN) metabolic pathway is proposed to be an emerging player in immunoregulatory networks *in vivo* due to its importance in mediating the equilibrium between activation and inhibition of the immune system (Mándi and Vécsei, 2012). TRP-KYN pathway is the primary metabolic pathway of tryptophan that is significantly activated by acute and chronic immune responses. Over 95% of TRP was catalyzed and transformed into a variety of metabolites, including KYN, kynurenic acid (KYNA), xanthurenic acid (XA) and cinnabarinic acid (CA) (Tanaka et al., 2021). These metabolites subsequently play a key role in modulating inflammation through positive or negative feedback loops and in the induction of immune tolerance. In this study, the level of kynurenine acid increased in the Model group. It indicated that more tryptophan is metabolized to form kynurenine acid during inflammation. After the intervention of gallic acid, the level of kynurenine acid decreased. It suggested that ellagic acid may help to restore the immune balance by regulating kynurenine metabolism.

## Conclusion

In the present work, we investigated the regulating effect of gallic acid on the downstream metabolic disorder in inflammation. A characteristic metabolic profile including 12 metabolic markers was identified in acute inflammation with HPLC-MS-based metabolomics and multivariate data analysis. These potential metabolites are mainly involved in linoleic acid metabolism, ascorbate and aldarate metabolism, pentose and glucuronate interconversions, and arachidonic acid metabolism pathways. The metabolic pathways analysis revealed that gallic acid could exert its anti-inflammatory effect by increasing antioxidant capacity, regulating lipid metabolism, alleviating the amplification of inflammatory cascades, inhibiting the proliferation of immune cells, and promoting the immune balance. These results could provide a theoretical basis for clarifying the mechanism of the anti-inflammatory effect of gallic acid.

## Data Availability

The original contributions presented in the study are included in the article/Supplementary Material, further inquiries can be directed to the corresponding author.

## References

[B1] BenSaad L. A. Kim K. H. Quah C. C. Kim W. R. Shahimi M. (2017). Anti-Inflammatory Potential of Ellagic Acid, Gallic Acid and Punicalagin A&B Isolated from Punica Granatum. BMC Complement. Altern. Med. 17, 47. 10.1186/s12906-017-1555-0 28088220 PMC5237561

[B2] Borges A. Ferreira C. Saavedra M. J. Simões M. (2013). Antibacterial Activity and Mode of Action of Ferulic and Gallic Acids against Pathogenic Bacteria. Microb. Drug Resist. 19, 256–265. 10.1089/mdr.2012.0244 23480526

[B3] Calder P. C. (2006). n-3 Polyunsaturated Fatty Acids, Inflammation, and Inflammatory Diseases. Am. J. Clin. Nutr. 83, 1505s–1519s. 10.1093/ajcn/83.6.1505S 16841861

[B4] Dehghani M. A. Shakiba Maram N. Moghimipour E. Khorsandi L. Atefi Khah M. Mahdavinia M. (2020). Protective Effect of Gallic Acid and Gallic Acid-Loaded Eudragit-RS 100 Nanoparticles on Cisplatin-Induced Mitochondrial Dysfunction and Inflammation in Rat Kidney. Biochim. Biophys. Acta Mol. Basis Dis. 1866, 165911. 10.1016/j.bbadis.2020.165911 32768679

[B5] Dong R. Tian Q. Shi Y. Chen S. Zhang Y. Deng Z. (2021). An Integrated Strategy for Rapid Discovery and Identification of Quality Markers in Gardenia Fructus Using an Omics Discrimination-Grey Correlation-Biological Verification Method. Front. Pharmacol. 12, 705498. 10.3389/fphar.2021.705498 34248647 PMC8264552

[B6] Fiehn O. Kopka J. Dörmann P. Altmann T. Trethewey R. N. Willmitzer L. (2000). Metabolite Profiling for Plant Functional Genomics. Nat. Biotechnol. 18, 1157–1161. 10.1038/81137 11062433

[B7] Gwon M. H. Yun J. M. (2021). Phenethyl Isothiocyanate Improves Lipid Metabolism and Inflammation via mTOR/PPARγ/AMPK Signaling in the Adipose Tissue of Obese Mice. J. Med. Food 24, 666–669. 10.1089/jmf.2020.4881 34077672

[B8] Hsieh S. C. Wu C. H. Wu C. C. Yen J. H. Liu M. C. Hsueh C. M. (2014). Gallic Acid Selectively Induces the Necrosis of Activated Hepatic Stellate Cells via a Calcium-Dependent Calpain I Activation Pathway. Life Sci. 102, 55–64. 10.1016/j.lfs.2014.02.041 24631138

[B9] Hunter M. Wang Y. Eubank T. Baran C. Nana-Sinkam P. Marsh C. (2009). Survival of Monocytes and Macrophages and Their Role in Health and Disease. Front. Biosci. (Landmark Ed) 14, 4079–4102. 10.2741/3514 19273336 PMC3708298

[B10] Huo T. Chen X. Lu X. Qu L. Liu Y. Cai S. (2014). An Effective Assessment of Valproate Sodium-Induced Hepatotoxicity with UPLC-MS and (1)HNMR-Based Metabonomics Approach. J. Chromatogr. B Analyt Technol. Biomed. Life Sci. 969, 109–116. 10.1016/j.jchromb.2014.08.011 25168794

[B11] Jin L. Sun S. Ryu Y. Piao Z. H. Liu B. Choi S. Y. (2018). Gallic Acid Improves Cardiac Dysfunction and Fibrosis in Pressure Overload-Induced Heart Failure. Sci. Rep. 8, 9302. 10.1038/s41598-018-27599-4 29915390 PMC6006337

[B12] Kianian F. Karimian S. M. Kadkhodaee M. Takzaree N. Seifi B. Adeli S. (2019). Combination of Ascorbic Acid and Calcitriol Attenuates Chronic Asthma Disease by Reductions in Oxidative Stress and Inflammation. Respir. Physiol. Neurobiol. 270, 103265. 10.1016/j.resp.2019.103265 31404684

[B13] Kubala L. Schmelzer K. R. Klinke A. Kolarova H. Baldus S. Hammock B. D. (2010). Modulation of Arachidonic and Linoleic Acid Metabolites in Myeloperoxidase-Deficient Mice during Acute Inflammation. Free Radic. Biol. Med. 48, 1311–1320. 10.1016/j.freeradbiomed.2010.02.010 20156554 PMC2856720

[B14] Lambert J. D. Elias R. J. (2010). The Antioxidant and Pro-Oxidant Activities of Green Tea Polyphenols: A Role in Cancer Prevention. Arch. Biochem. Biophys. 501, 65–72. 10.1016/j.abb.2010.06.013 20558130 PMC2946098

[B15] Lei M. Tao M. Q. Wu Y. J. Xu L. Yang Z. Li Y. (2021). Metabolic Enzyme Triosephosphate Isomerase 1 and Nicotinamide Phosphoribosyltransferase, Two Independent Inflammatory Indicators in Rheumatoid Arthritis: Evidences from Collagen-Induced Arthritis and Clinical Samples. Front. Immunol. 12, 795626. 10.3389/fimmu.2021.795626 35111160 PMC8801790

[B16] Liu H. Zhang L. Zhao B. Zhang Z. Qin L. Zhang Q. (2015). Hypothalamus Metabolomic Profiling to Elucidate the Tissue-Targeted Biochemical Basis of Febrile Response in Yeast-Induced Pyrexia Rats. Chem. Biol. Interact 231, 61–70. 10.1016/j.cbi.2015.02.018 25746356

[B17] Liu Y. L. Hsu C. C. Huang H. J. Chang C. J. Sun S. H. Lin A. M. (2020). Gallic Acid Attenuated LPS-Induced Neuroinflammation: Protein Aggregation and Necroptosis. Mol. Neurobiol. 57, 96–104. 10.1007/s12035-019-01759-7 31832973

[B18] Ma N. Yang Y. Liu X. Li S. Qin Z. Li J. (2020). Plasma Metabonomics and Proteomics Studies on the Anti-Thrombosis Mechanism of Aspirin Eugenol Ester in Rat Tail Thrombosis Model. J. Proteomics 215, 103631. 10.1016/j.jprot.2019.103631 31891783

[B19] Mahnashi M. H. Alyami B. A. Alqahtani Y. S. Jan M. S. Rashid U. Sadiq A. (2021). Phytochemical Profiling of Bioactive Compounds, Anti-Inflammatory and Analgesic Potentials of Habenaria Digitata Lindl.: Molecular Docking Based Synergistic Effect of the Identified Compounds. J. Ethnopharmacol 273, 113976. 10.1016/j.jep.2021.113976 33647424

[B20] Mándi Y. Vécsei L. (2012). The Kynurenine System and Immunoregulation. J. Neural Transm. (Vienna) 119, 197–209. 10.1007/s00702-011-0681-y 21744051

[B21] Monteiro J. Askarian F. Nakamura M. T. Moghadasian M. H. Ma D. W. (2013). Oils Rich in α-Linolenic Acid Independently Protect against Characteristics of Fatty Liver Disease in the Δ6-Desaturase Null Mouse. Can. J. Physiol. Pharmacol. 91, 469–479. 10.1139/cjpp-2012-0308 23746194

[B22] Morris C. J. (2003). Carrageenan-Induced Paw Edema in the Rat and Mouse. Methods Mol. Biol. 225, 115–121. 10.1385/1-59259-374-7:115 12769480

[B23] Nam B. Rho J. K. Shin D. M. Son J. (2016). Gallic Acid Induces Apoptosis in EGFR-Mutant Non-Small Cell Lung Cancers by Accelerating EGFR Turnover. Bioorg. Med. Chem. Lett. 26, 4571–4575. 10.1016/j.bmcl.2016.08.083 27597244

[B24] Olsson A. Gustavsen S. Nguyen T. D. Nyman M. Langkilde A. R. Hansen T. H. (2021). Serum Short-Chain Fatty Acids and Associations with Inflammation in Newly Diagnosed Patients with Multiple Sclerosis and Healthy Controls. Front. Immunol. 12, 661493. 10.3389/fimmu.2021.661493 34025661 PMC8134701

[B25] Pandurangan A. K. Mohebali N. Esa N. M. Looi C. Y. Ismail S. Saadatdoust Z. (2015). Gallic Acid Suppresses Inflammation in Dextran Sodium Sulfate-Induced Colitis in Mice: Possible Mechanisms. Int. Immunopharmacol 28, 1034–1043. 10.1016/j.intimp.2015.08.019 26319951

[B26] Pang J. S. Yen J. H. Wu H. T. Huang S. T. (2017). Gallic Acid Inhibited Matrix Invasion and AP-1/ETS-1-Mediated MMP-1 Transcription in Human Nasopharyngeal Carcinoma Cells. Int. J. Mol. Sci. 18 (7), 1354. 10.3390/ijms18071354 PMC5535847 28672814

[B27] Pereira R. R. Amladi S. T. Varthakavi P. K. (2011). A Study of the Prevalence of Diabetes, Insulin Resistance, Lipid Abnormalities, and Cardiovascular Risk Factors in Patients with Chronic Plaque Psoriasis. Indian J. Dermatol. 56, 520–526. 10.4103/0019-5154.87144 22121269 PMC3221214

[B28] Priscilla D. H. Prince P. S. (2009). Cardioprotective Effect of Gallic Acid on Cardiac Troponin-T, Cardiac Marker Enzymes, Lipid Peroxidation Products and Antioxidants in Experimentally Induced Myocardial Infarction in Wistar Rats. Chem. Biol. Interact 179, 118–124. 10.1016/j.cbi.2008.12.012 19146839

[B29] Rahimifard M. Baeeri M. Bahadar H. Moini-Nodeh S. Khalid M. Haghi-Aminjan H. (2020). Therapeutic Effects of Gallic Acid in Regulating Senescence and Diabetes; an *In Vitro* Study. Molecules 25 (24), 5875. 10.3390/molecules25245875 PMC7763304 33322612

[B30] Rasool M. K. Sabina E. P. Ramya S. R. Preety P. Patel S. Mandal N. (2010). Hepatoprotective and Antioxidant Effects of Gallic Acid in Paracetamol-Induced Liver Damage in Mice. J. Pharm. Pharmacol. 62, 638–643. 10.1211/jpp.62.05.0012 20609067

[B31] Reyes A. W. B. Arayan L. T. Hop H. T. Ngoc Huy T. X. Vu S. H. Min W. (2018). Effects of Gallic Acid on Signaling Kinases in Murine Macrophages and Immune Modulation against Brucella Abortus 544 Infection in Mice. Microb. Pathog. 119, 255–259. 10.1016/j.micpath.2018.04.032 29680683

[B32] Seo C. S. Jeong S. J. Yoo S. R. Lee N. R. Shin H. K. (2016). Quantitative Analysis and *In Vitro* Anti-Inflammatory Effects of Gallic Acid, Ellagic Acid, and Quercetin from Radix Sanguisorbae. Pharmacogn Mag. 12, 104–108. 10.4103/0973-1296.177908 27076745 PMC4809163

[B33] Setayesh T. Nersesyan A. Mišík M. Noorizadeh R. Haslinger E. Javaheri T. (2019). Gallic Acid, a Common Dietary Phenolic Protects against High Fat Diet Induced DNA Damage. Eur. J. Nutr. 58, 2315–2326. 10.1007/s00394-018-1782-2 30039436 PMC6689278

[B34] Shui S. Shen S. Huang R. Xiao B. Yang J. (2016). Metabonomic Analysis of Biochemical Changes in the Plasma and Urine of Carrageenan-Induced Rats after Treatment with Yi-Guan-Jian Decoction. J. Chromatogr. B Analyt Technol. Biomed. Life Sci. 1033-1034, 80–90. 10.1016/j.jchromb.2016.08.003 27525358

[B35] Singla E. Dharwal V. Naura A. S. (2020). Gallic Acid Protects against the COPD-Linked Lung Inflammation and Emphysema in Mice. Inflamm. Res. 69, 423–434. 10.1007/s00011-020-01333-1 32144443

[B36] Singla E. Puri G. Dharwal V. Naura A. S. (2021). Gallic Acid Ameliorates COPD-Associated Exacerbation in Mice. Mol. Cel Biochem 476, 293–302. 10.1007/s11010-020-03905-5 32965595

[B37] Sripanidkulchai B. Junlatat J. (2014). Bioactivities of Alcohol Based Extracts of Phyllanthus Emblica Branches: Antioxidation, Antimelanogenesis and Anti-Inflammation. J. Nat. Med. 68, 615–622. 10.1007/s11418-014-0824-1 24557876

[B38] Tanaka M. Sugama A. Sumi K. Shimizu K. Kishimoto Y. Kondo K. (2020). Gallic Acid Regulates Adipocyte Hypertrophy and Suppresses Inflammatory Gene Expression Induced by the Paracrine Interaction between Adipocytes and Macrophages *In Vitro* and *In Vivo* . Nutr. Res. 73, 58–66. 10.1016/j.nutres.2019.09.007 31841748

[B39] Tanaka M. Toth F. Polyak H. Szabo A. Mandi Y. Vecsei L. (2021). Immune Influencers in Action: Metabolites and Enzymes of the Tryptophan-Kynurenine Metabolic Pathway. Biomedicines 9 (7), 734. 10.3390/biomedicines9070734 34202246 PMC8301407

[B40] van der Greef J. Stroobant P. van der Heijden R. (2004). The Role of Analytical Sciences in Medical Systems Biology. Curr. Opin. Chem. Biol. 8, 559–565. 10.1016/j.cbpa.2004.08.013 15450501

[B41] Verma S. Singh A. Mishra A. (2013). Gallic Acid: Molecular Rival of Cancer. Environ. Toxicol. Pharmacol. 35, 473–485. 10.1016/j.etap.2013.02.011 23501608

[B42] Wang M. Huang J. Fan H. He D. Zhao S. Shu Y. (2018). Treatment of Rheumatoid Arthritis Using Combination of Methotrexate and Tripterygium Glycosides Tablets-A Quantitative Plasma Pharmacochemical and Pseudotargeted Metabolomic Approach. Front. Pharmacol. 9, 1051. 10.3389/fphar.2018.01051 30356765 PMC6189563

[B43] Wang T. Fu X. Chen Q. Patra J. K. Wang D. Wang Z. (2019). Arachidonic Acid Metabolism and Kidney Inflammation. Int. J. Mol. Sci. 20 (15), 3683. 10.3390/ijms20153683 PMC6695795 31357612

[B44] Wang X. Hunter D. Xu J. Ding C. (2015). Metabolic Triggered Inflammation in Osteoarthritis. Osteoarthritis Cartilage 23, 22–30. 10.1016/j.joca.2014.10.002 25452156

[B45] Wei G. Wu Y. Gao Q. Shen C. Chen Z. Wang K. (2018). Gallic Acid Attenuates Postoperative Intra-Abdominal Adhesion by Inhibiting Inflammatory Reaction in a Rat Model. Med. Sci. Monit. 24, 827–838. 10.12659/MSM.908550 29429982 PMC5815494

[B46] Wenzl F. A. Ambrosini S. Mohammed S. A. Kraler S. Lüscher T. F. Costantino S. (2021). Inflammation in Metabolic Cardiomyopathy. Front. Cardiovasc. Med. 8, 742178. 10.3389/fcvm.2021.742178 34671656 PMC8520939

[B47] Yao W. Zhang L. Hua Y. Ji P. Li P. Li J. (2015). The Investigation of Anti-Inflammatory Activity of Volatile Oil of Angelica Sinensis by Plasma Metabolomics Approach. Int. Immunopharmacol 29, 269–277. 10.1016/j.intimp.2015.11.006 26578286

[B48] Yara S. Lavoie J. C. Beaulieu J. F. Delvin E. Amre D. Marcil V. (2013). Iron-Ascorbate-Mediated Lipid Peroxidation Causes Epigenetic Changes in the Antioxidant Defense in Intestinal Epithelial Cells: Impact on Inflammation. PLoS One 8, e63456. 10.1371/journal.pone.0063456 23717425 PMC3661745

[B49] Yoon C. H. Chung S. J. Lee S. W. Park Y. B. Lee S. K. Park M. C. (2013). Gallic Acid, a Natural Polyphenolic Acid, Induces Apoptosis and Inhibits Proinflammatory Gene Expressions in Rheumatoid Arthritis Fibroblast-Like Synoviocytes. Jt. Bone Spine 80, 274–279. 10.1016/j.jbspin.2012.08.010 23058179

[B50] Zhang J. Li X. Wei J. Chen H. Lu Y. Li L. (2018). Gallic Acid Inhibits the Expression of Keratin 16 and Keratin 17 through Nrf2 in Psoriasis-Like Skin Disease. Int. Immunopharmacol 65, 84–95. 10.1016/j.intimp.2018.09.048 30293051

[B51] Zhu L. Gu P. Shen H. (2019). Gallic Acid Improved Inflammation via NF-κB Pathway in TNBS-Induced Ulcerative Colitis. Int. Immunopharmacol 67, 129–137. 10.1016/j.intimp.2018.11.049 30544066

[B52] Zhuo Q. Yang W. Chen J. Wang Y. (2012). Metabolic Syndrome Meets Osteoarthritis. Nat. Rev. Rheumatol. 8, 729–737. 10.1038/nrrheum.2012.135 22907293

